# Editorial: Fast Multi-Parameter Magnetic Resonance Neuroimaging

**DOI:** 10.3389/fnins.2022.948993

**Published:** 2022-06-29

**Authors:** Jiazheng Wang, Shuhui Cai, Zhiyong Zhang, Congbo Cai

**Affiliations:** ^1^Clinical & Technical Support, Philips Healthcare, Beijing, China; ^2^School of Electronic Science and Technology, Xiamen University, Xiamen, China; ^3^School of Biomedical Engineering, Shanghai Jiao Tong University, Shanghai, China

**Keywords:** multi-parametric, neuroimaging, oxygen extraction fraction, magnetization transfer, chemical exchange saturation transfer, diffusion tensor imaging

Multiparametric MRI has been extensively studied in prostate imaging, showing diagnostic benefits with high-quality evidence. In 2019, the PI-RADS Steering Committee confirmed that multiparametric MRI helps to distinguish patients with clinically insignificant cancer from those with life-threatening cancer, and the Committee suggested multiparametric MRI routine adoption in clinical practice (Padhani et al., 2019). On the other hand, despite the fast development in the past two decades, multiparametric MRI in the brain has not established a solid role in the clinic. In theory, multiparametric MRI allows for the detection of microstructural processes in the tissue impairment or remodeling in neuro diseases, which could provide added values to conventional MRI that is restricted to macroscopic tissue pathology. However, the clinical application of multiparametric MRI is still dependent on the solution of a series of challenges, including the relatively long acquisition time, lack of histological validations (Seiler et al., 2021), lack of standardized imaging sequences and protocols (Sawlani et al., 2020), and the concerns in repeatability and reproducibility (Keenan et al., 2022). In this Research Topic “Fast Multi-Parameter Magnetic Resonance Neuroimaging”, researchers aimed to answer some of these questions.

On cancerous neuro disease, Shen et al. exploited Oxygen Extraction Fraction (OEF) mapping for the differential diagnosis of gliomas between a series of genome types. The OEF mapping was calculated with an integration of both quantitative susceptibility mapping (QSM) and quantitative blood oxygen level-dependent magnitude (qBOLD). As a biomarker predominantly reflecting the hypoxia status, the OEF mappings achieved the AUCs of 0.828, 0.784, and 0.764 for the detection of IDH1 mutation in low-grade gliomas, MGMT promoter methylation in glioblastomas, and RTK aberration in the general cohort, respectively (with *p*-values of < 0.001, 0.001, and 0.005, respectively). With a cohort of 91 patients with pathologically confirmed gliomas, these results suggest the potential of OEF mapping in gliomas differential diagnosis and treatment guidance.

On the other side of the disease spectrum, Bao et al., Kisel et al., and Liu et al. focused on the application of multiparametric MRI in neurodegenerative diseases. When compared to the age-matched healthy controls, the remitting-relapsing multiple sclerosis (RRMS) patients demonstrated significantly different patterns of fractional anisotropy, mean diffusivity, axial diffusivity, and radial diffusivity in a range of brain areas, as revealed by Bao et al. using diffusion tensor imaging, suggesting demyelination and axonal injury in RRMS patients even in some normal-appearing white matter regions. Kisel et al. reviewed the value of Macromolecular Proton Fraction (MPF) measurement, using magnetization transfer (MT) MRI, in the observation of the de- and re-myelination process. This work demonstrated the animal studies that formed the basis of MPF mapping theory, the correlation of MPF maps with histological studies, and the recent translation of this technique at clinical setups, suggesting the potential application of MPF mapping in a range of neurodegenerative diseases including MS by providing additional pathological information in a non-invasive way. Meanwhile, Liu et al. applied chemical exchange saturation transfer (CEST) MRI to Alzheimer's disease (AD), revealing higher glutamate concentration in AD mice than in wild type of the same age. With a Resnet neural network, they were able to achieve an accuracy of 75.6% in the classification of glutamate distribution images of AD mice and the wild type. Their result implies a potential solution to AD diagnosis comprising both novel MRI techniques such as CEST and the recent development in artificial intelligence (AI).

With incremental clinical values revealed and validated by histological studies and with integrated development in both acquisition technology and AI-based image interpretation, the studies in this Research Topic have shed light on the potential clinical impacts of multiparametric MRI. However, before being translated into clinical practice, several questions mentioned above remain unanswered. A particularly important concern is the repeatability and reproducibility of multiparametric MRI, which is a legacy problem that has for a long time hampered the clinical translation of quantitative MRI. Is the multiparametric MRI-based diagnosis model more prone to the across-site and across-vendor signal variations, even assuming the same imaging sequence and protocol, or is it more robust than traditional quantitative MRI because of the integration of different measured parameters? In addition, the standardization of acquisition sequence and protocols is another major challenge for the routine use of multiparametric MRI. Nevertheless, any progress in multiparametric MRI toward clinical translation will be plausible to the modern healthcare system due to its remarkable cost-benefit and reduced operational risk when compared to the traditional biopsy-based tissue examination.

## Author Contributions

JW wrote the manuscript. All authors critically reviewed and approved the manuscript. All authors contributed to the article and approved the submitted version.

## Conflict of Interest

JW is an employee of Philips Healthcare. The remaining authors declare that the research was conducted in the absence of any commercial or financial relationships that could be construed as a potential conflict of interest.

## Publisher's Note

All claims expressed in this article are solely those of the authors and do not necessarily represent those of their affiliated organizations, or those of the publisher, the editors and the reviewers. Any product that may be evaluated in this article, or claim that may be made by its manufacturer, is not guaranteed or endorsed by the publisher.
